# BAG3 Protein Is Involved in Endothelial Cell Response to Phenethyl Isothiocyanate

**DOI:** 10.1155/2018/5967890

**Published:** 2018-05-31

**Authors:** Silvia Franceschelli, Anna Paola Bruno, Michela Festa, Antonia Falco, Elisa Gionti, Morena d'Avenia, Margot De Marco, Anna Basile, Vittoria Iorio, Liberato Marzullo, Alessandra Rosati, Maria Pascale

**Affiliations:** ^1^Department of Pharmacy (DIFARMA), University of Salerno, Via Giovanni Paolo II 132, 84084 Fisciano, Italy; ^2^Department of Molecular Medicine and Medical Biotechnology, University of Naples Federico II, Via Pansini 5, 80131 Naples, Italy; ^3^Department of Medicine, Surgery and Dentistry “Scuola Medica Salernitana”, University of Salerno, Via S. Allende, 84081 Baronissi, Italy

## Abstract

Phenethyl isothiocyanate (PEITC), a cruciferous vegetable-derived compound, is a versatile cancer chemopreventive agent that displays the ability to inhibit tumor growth during initiation, promotion, and progression phases in several animal models of carcinogenesis. In this report, we dissect the cellular events induced by noncytotoxic concentrations of PEITC in human umbilical vein endothelial cells (HUVECs). In the early phase, PEITC treatment elicited cells' morphological changes that comprise reduction in cell volume and modification of actin organization concomitantly with a rapid activation of the PI3K/Akt pathway. Downstream to PI3K, PEITC also induces the activity of Rac1 and activation of c-Jun N-terminal kinase (JNK), well-known regulators of actin cytoskeleton dynamics. Interestingly, PEITC modifications of the actin cytoskeleton were abrogated by pretreatment with JNK inhibitor, SP600125. JNK signaling led also to the activation of the c-Jun transcription factor, which is involved in the upregulation of several genes; among them is the BAG3 protein. This protein, a member of the BAG family of heat shock protein (Hsp) 70 cochaperones, is able to sustain survival in different tumor cell lines and neoangiogenesis by directly regulating the endothelial cell cycle. Furthermore, BAG3 is involved in maintaining actin folding. Our findings indicate that BAG3 protein expression is induced in endothelial cells upon exposure to a noncytotoxic concentration of PEITC and its expression is requested for the recovery of normal cell size and morphology after the stressful stimuli. This assigns an additional role for BAG3 protein in the endothelial cells after a stress event.

## 1. Introduction

Several epidemiologic studies support the hypothesis that dietary intake of cruciferous vegetables may have protective effects against the risk of different types of cancers [1–4]. Chemopreventive and anticarcinogenic effects of cruciferous vegetables are attributed to organic isothiocyanates (ITCs), which naturally occur in a variety of edible cruciferous vegetables such as broccoli, watercress, and cabbage in which they are stored as glucosinolate precursors [5]. ITCs are effective in blocking carcinogenesis in a variety of tissues and are known to inhibit angiogenesis in vitro and in vivo [6–8].

Phenethyl isothiocyanate (PEITC) is one of the best-studied members of the ITC family, due to its anticarcinogenic and antiangiogenic activities reported in myelomas [7] and lung [9] and prostate cancer [10, 11].

In particular, the antiangiogenic properties of PEITC may be largely related to suppression of vascular endothelial growth factor (VEGF) secretion, downregulation of vascular endothelial growth factor receptor-2 protein (VEGF-R2), and Akt inactivation [12, 13].

Furthermore, several studies indicate that ITCs can modulate the expression level of hypoxia-inducible factors (HIF) in tumors [14] and endothelial cells [15]; in fact, PEITC inhibits HIF transcription [16]. Induction of HIF in hypoxic conditions increases the level of proangiogenic factors, including interleukin 8 (IL8), angiopoietin 2 (Ang2), and VEGF. Moreover, the decreased translational efficiency of the HIF1*α* subunit may contribute to the antiangiogenetic effect of PEITC [7]. PEITC can also induce cellular oxidative stress by rapidly conjugating glutathione (GSH). Depletion of GSH followed by rapid accumulation of ROS may be related to PEITC-mediated apoptotic cell death [9, 17]. An additional more interesting property of PEITC and sulforaphane (SFN) regards the ability to determine disruption of microtubule polymerization *in vitro* and *in vivo*. In fact, PEITC can form covalent binding with microtubular tubulin, leading to the collapse of the microtubule cytoskeleton in A549 cells. This property may explain the ITC-induced mitotic cell cycle arrest and apoptosis observed in cancer and noncancer cells [13, 18]. It is of interest that BAG3, the only stress-inducible member of the BAG family of cochaperones, has been reported to sensitize HPV18+ HeLa cells to PEITC-induced apoptosis restoring p53 levels [19]. Indeed, BAG3 has also been described for its ability to sustain neoangiogenesis acting on the endothelial cell cycle [20] and to regulate actin folding through its interaction with the cytosolic chaperonin CCT (containing TCP-1) [21].

In this study, we report for the first time that the F-actin cytoskeleton undergoes remodeling during the early phase of PEITC treatment of HUVECs. Our data indicate that this effect is associated with an immediate activation of the PI3K/Akt and PI3K/Rac1/JNK pathways. Pretreatment with the JNK inhibitor SP600125 reverses PEITC-induced actin filament remodeling, thus providing direct evidence of JNK-mediated actin cytoskeleton modifications occurring immediately after PEITC administration. The stress-induced BAG3 protein was found increased in its levels upon PEITC induction. We also found a marked colocalization of BAG3 with F-actin, thus sustaining its role in actin localization and cellular morphology changes upon PEITC treatment.

## 2. Results

### 2.1. PEITC Induces Actin Cytoskeleton Alterations and Activation of the PI3K/Akt Pathway in HUVECs

**Figure 1:**
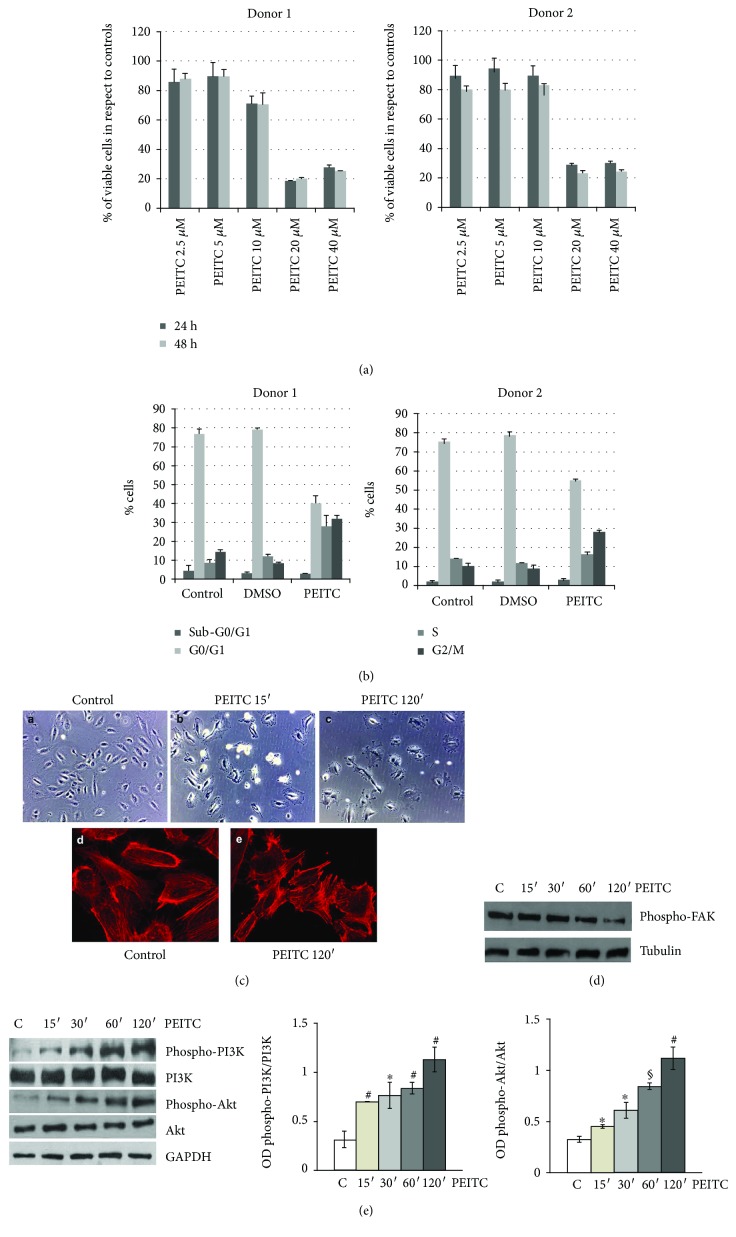
PEITC induces actin cytoskeleton alterations and activation of the PI3K/Akt pathway in HUVECs. (a) HUVECs from 2 different donors were plated at 5 ×10^3^/cm^2^ and then treated with PEITC at indicated concentrations for 24 and 48 h. Then, cells were subjected to the MTT assay, and results are shown in a bar graph as % of viable cells in respect to controls (untreated or 0.01% DMSO-treated cells). (b) HUVECs from 2 different donors were treated as described above. After 24 h, a cell cycle assay was performed by using PI on permeabilized cells. Results are expressed as % of cells in each cell cycle phase. (c) Phase-contrast images and confocal analysis of HUVECs treated with (A, D) DMSO (control) at a final concentration (0.01%), with (B) PEITC (10 *μ*M) for 15 min, and with (C, E) PEITC (10 *μ*M) for 120 min. (B, C, E) Images of PEITC-treated cells showing blebbing formation in the outer cytoskeletal domain. (d) HUVECs were seeded as described above and treated with PEITC; at the indicated time points, cells were harvested and subjected to cell lysis. Protein contents were analyzed by Western blot to analyze the levels of phospho-FAK protein. Tubulin was used as a loading control. (e) HUVECs were treated with 0.01% DMSO (C) and with 10 *μ*M PEITC for 15, 30, 60, and 120 min. Total protein extracts were analyzed by Western blot using anti-phospho-Akt (Ser473), anti-Akt, anti-phospho-PI3K, and anti-PI3K to test PI3K/Akt activation levels. The anti-GAPDH antibody was used as an internal loading control. Each lane was loaded with 20 *μ*g protein. The bar graph depicts densitometric analysis of the data (expressed as phospho-PI3K/PI3K and phospho-Akt/Akt ratios) corresponding to the left panel. Results were obtained from at least two independent experiments and are expressed as the mean ± SEM. ^∗^*P* < 0.05, ^#^*P* < 0.01, and ^§^*P* < 0.001, statistically significant differences, compared to DMSO-treated cells (C), were calculated by Student's *t*-test for unpaired data.

Human endothelial vein umbilical cells are a well-known model used to study many aspects of endothelial function and disease, such as normal, abnormal, and tumor-associated angiogenesis, oxidative stress, and hypoxia-related pathways in endothelia under normal and pathological conditions. We have observed a cytotoxic effect of PEITC at 20 and 40 *μ*M concentrations as revealed by an MTT assay performed on two different HUVEC donors. In particular, we observed a decrease of more than 75% of the viable cells in respect to controls at a concentration of 20 *μ*M after 24 and 48 h (Figure 1(a)), in contrast with evidences published by Xiao and Singh [22] which demonstrate a concentration of 2 *μ*M as cytotoxic. Indeed, in our hands, the treatment of HUVECs at 10 *μ*M PEITC was not cytotoxic, but we observed an effect on the cell cycle as shown in Figure 1(b); in HUVECs from two different donors, we observed, at 24 hours, cells accumulating in S and G2/M phases. However, this effect on the cell cycle does not significantly affect the cell number after 48 hours of treatment in respect to 24 hours (Figure 1(a)), thus suggesting that cells have activated survival pathways to overcome the PEITC insult. Phase-contrast observations of HUVECs revealed that PEITC induced a dramatic change in cellular morphology after 15 and 120 min of treatment (Figure 1(c), A–C). Furthermore, control HUVECs show a highly developed actin cytoskeleton with clearly visible, numerous stress fibers (Figure 1(c), D). After 120 min treatment with PEITC, we observed distinct changes in cell morphology and reorganization of actin filaments (Figure 1(c), E). Cells reduce their volume, which resulted in a decrease in the area of intercellular contacts (Figure 1(c), B–E). Phalloidin staining shows the disappearance of actin stress fibers and short fibril or small aggregate formations. Focal adhesion kinase (FAK) has been widely reported to be involved in the control of focal adhesion organization and stress fiber formation, through phosphorylation and dephosphorylation of its tyrosine residues [23]. Therefore, we further investigated whether PEITC treatment modulates FAK phosphorylation; Figure 1(d) shows a decrease in FAK phosphorylation after treatment with 10 *μ*M of PEITC for 120 min.

In cancer cells, PEITC is known to downregulate the PI3K/Akt prosurvival pathway through a marked decrease in Akt Ser473 phosphorylation and subsequent apoptosis [13] and the involvement of the PI3K/Akt pathway in actin filament remodeling and cell migration is well known [24–26]. In order to verify, in the endothelial primary cell model, a possible connection between Akt activity and the effect on actin remodeling upon PEITC treatment, we first focused on PI3K and Akt phosphorylation/activation kinetics. As shown in Figure 1(e), cell exposure to 10 *μ*M PEITC for 15, 30, 60, and 120 min revealed that levels of phospho-Akt and phospho-PI3K started to increase as early as 15 min, the highest levels being obtained 2 h after PEITC administration. No significant changes occurred in the expression of total PI3K and Akt. These results indicated that high activation of the PI3K/Akt prosurvival pathway can be observed concomitantly with cell morphology and actin redistribution changes.

### 2.2. PEITC Induces Rac1 Activity via PI3K, and Rac1 in turn Activates JNK

**Figure 2:**
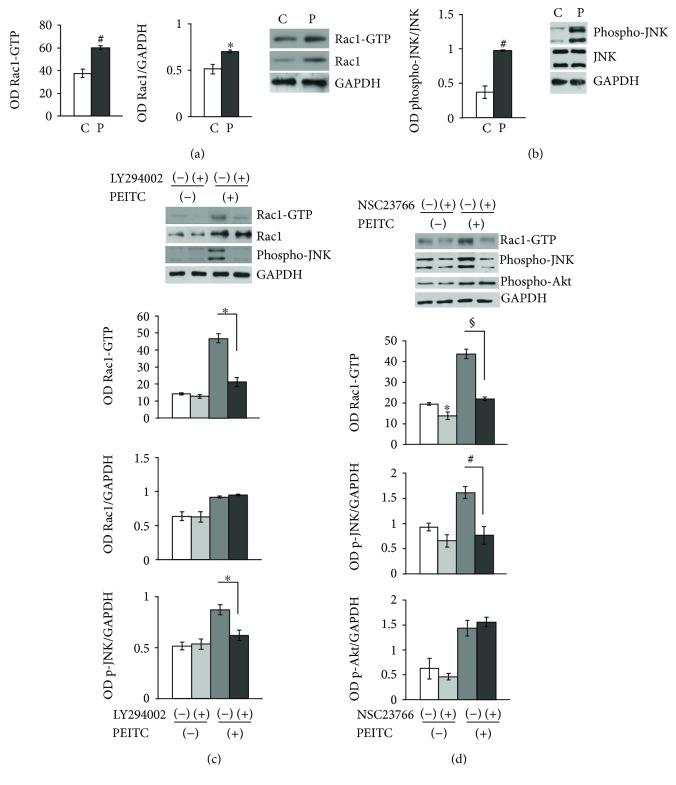
PEITC induces Rac1 activity via PI3K, and Rac1 in turn activates JNK. (a) HUVECs were treated with 0.01% DMSO (C) or treated with 10 *μ*M of PEITC (P) for 2 h and subjected to Western blot analysis of total proteins to measure the effect of PEITC on Rac1-GTPase activity and Rac1 total protein levels and on JNK activation. The left and middle panels display columns representing densitometric analysis of Rac1 activity and of Rac1 total protein levels (expressed as Rac1/GAPDH ratios). Representative blot analysis for Rac1 after a pull-down assay and for total Rac1 protein. (b) HUVECs were treated as described above. Total cell protein content was subjected to Western blot analysis to measure phospho-JNK levels. The left panel displays columns representing densitometric analysis of the data (expressed as phospho-JNK/JNK ratios) corresponding to the right panel. The anti-GAPDH antibody was used as an internal loading control. (c) The levels of GTP-bound Rac1, total Rac1, and phospho-JNK in lysates of pretreated or not with 10 *μ*M of LY2940021 (1 h) were analyzed in HUVECs in the presence or absence of 10 *μ*M PEITC stimulation for 2 h. The lower bar graphs depict densitometric analysis of Rac1 activity, Rac1 protein levels, and phospho-JNK levels. (d) The levels of GTP-bound Rac1, phospho-Akt, and phospho-JNK were analyzed in lysates of pretreated or not with 100 *μ*M of NSC23766 HUVECs for 4 h, in the presence/absence of 10 *μ*M PEITC stimulation for 2 h. The lower bar graphs depict densitometric analysis of Rac1 activity, phospho-JNK, and phospho-Akt levels. Results were obtained from at least two independent experiments and are expressed as the mean ± SEM. ^∗^*P* < 0.05, ^#^*P* < 0.01, and ^§^*P* < 0.001, statistically significant differences, compared to DMSO-treated cells (C), were calculated by Student's *t*-test for unpaired data.

**Figure 3:**
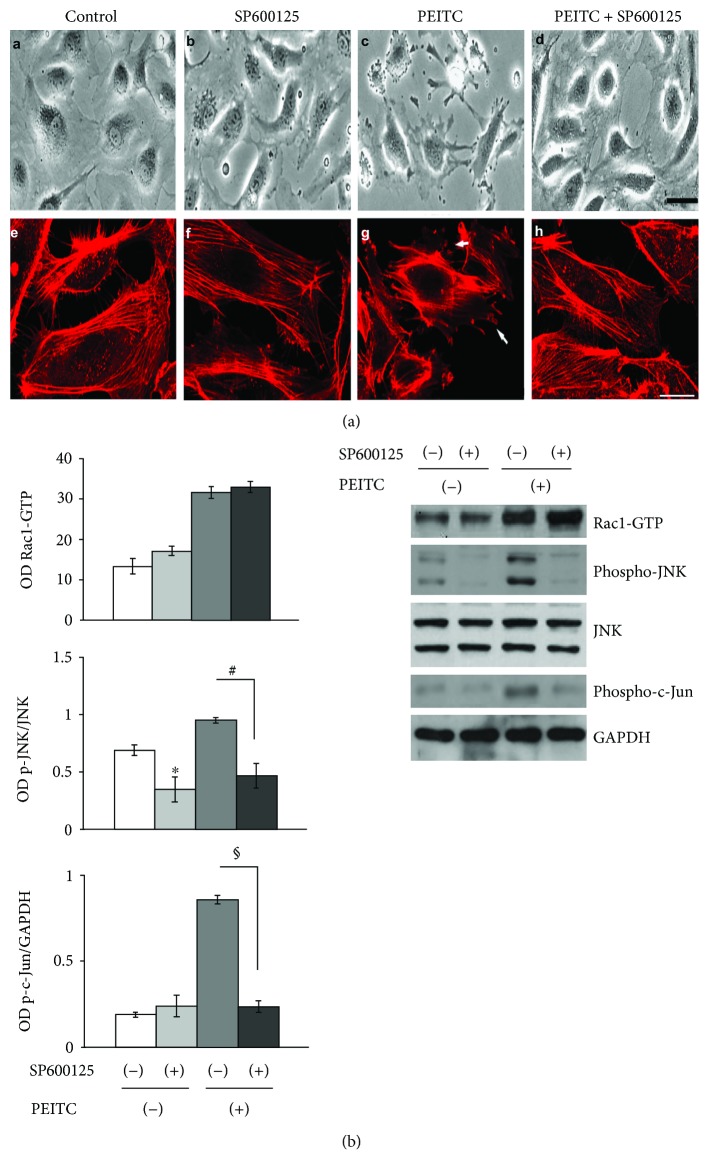
JNK activity is involved in actin remodeling processes during PEITC treatment. (a) The upper panel displays phase-contrast images and confocal analysis of HUVECs treated with (A, E) 0.01% DMSO (control), (B, F) 10 *μ*M of SP600125 (JNK inhibitor) for 1 h (C, G), and 10 *μ*M of PEITC for 2 h and (D, H) pretreated with 10 *μ*M of SP600125 1 h before PEITC administration. Scale bars: *d* = 15 *μ*m and *h* = 5 *μ*m. (b) The levels of GTP-bound Rac1, phospho-JNK, and phospho-c-Jun in lysates of HUVECs, pretreated or not with 10 *μ*M of SP600125 for 1 h, were analyzed in the presence/absence of 10 *μ*M PEITC stimulation for 2 h. The left bar graphs depict densitometric analysis of Rac1 activity, phospho-JNK, and phospho-c-JUN levels. Results were obtained from at least two independent experiments and are expressed as the mean ± SEM. ^∗^*P* < 0.05, ^#^*P* < 0.01, ^§^*P* < 0.001, statistically significant differences, compared to DMSO-treated cells (C), were calculated by Student's *t*-test for unpaired data.

PI3K and Rac1 are key molecules in the activation of cell migration brought about by dynamic changes in the actin cytoskeleton [27, 28]. PI3K is an important activator of Rac1 [28, 29]. Notably, in HUVECs, Rac1 is a downstream effector for PI3K but does not act upstream of Akt [30]. In addition, Rac1 activity is known to be dramatically reduced in HUVECs depleted of p110*α* (a class I PI 3-kinase catalytic subunit) [31]. A Rac1 activity assay was performed to investigate whether the protein is induced after PEITC treatment. As observed in Figure 2(a), an increase in Rac1 activity was present in 10 *μ*M PEITC-treated cells for 2 h compared to control cells. Unexpectedly, we also observed that PEITC induced an increase in total Rac1 protein, thus suggesting a novel mechanism for Rac1 upregulation. These results indicate that Rac1 probably acts as a downstream PI3K effector.

c-Jun N-terminal kinase (JNK, also known as stress-activated protein kinase-1 (SAPK1)) is strongly activated by cell stress inducers. UV irradiation, hyperosmolarity, and inflammatory cytokines stimulate the activity of JNK [32]. PEITC induces JNK activation in a dose-dependent manner [33]. To establish whether 10 *μ*M PEITC induced activation of JNK in our settings, Western blot analysis was performed and, as shown in Figure 2(b), PEITC elicited a significant increase in phospho-JNK activation after 2 h drug exposure. No effects on expression levels of total JNK were observed. These results suggest the involvement of JNK phosphorylation in PEITC-mediated remodeling of the actin filament cytoskeleton (Figure 3(a)).

To assess whether Rac1 is a downstream effector of PI3K in PEITC-treated HUVECs, we performed a pull-down assay in which GTP-bound Rac1 was isolated from control cells or cells pretreated for 1 h with 10 *μ*M LY294002, a PI3K inhibitor (p110*α* catalytic subunit inhibitor), in control or PEITC-treated cells. PI3K inhibition resulted in the concomitant downregulation of Rac1 activity and JNK phosphorylation levels as shown in Figure 2(c). Conversely, the PI3K inhibitor had no effect on a PEITC-mediated increase in total Rac1 protein levels. To investigate whether Rac1 acted upstream of JNK, HUVECs were cultured in the absence/presence of a specific Rac1-GEF inhibitor (NSC23766, 100 *μ*M) for 4 h and subsequently treated with PEITC for additional 2 h. As shown in Figure 2(d), inhibition of Rac1 downregulated JNK activation. In contrast, Rac1 did not seem to act upstream of Akt, since Rac1 inhibition by NSC23766 did not affect Akt phosphorylation in PEITC-treated cells. Taken together, these results suggest that the PI3K/Rac1/JNK pathway is activated in PEITC-treated endothelial cells exhibiting cytoskeleton modifications.

### 2.3. JNK Activity Is Involved in Actin Remodeling Processes during PEITC Treatment

**Figure 4:**
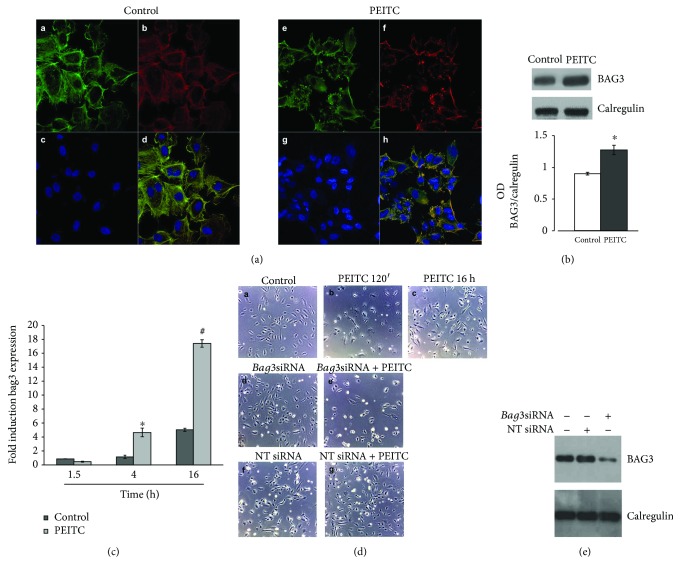
PEITC treatment induces BAG3 expression and its delocalization in HUVECs. (a) Immunofluorescence analysis of HUVECs in control conditions and after PEITC treatment. Cells were stained with the BAG3 antibody (A–E), phalloidin (B–F) that allows for the visualization of F-actin, and Hoechst (C–G), and D–H are merged images. The merged image shows overlapping localization of BAG3 and F-actin. (b) HUVECs were treated with 0.01% DMSO (control) and with 10 *μ*M PEITC for 16 h. Total protein extracts were analyzed by Western blot using the anti-BAG3 antibody and anti-calregulin antibody, as an internal loading control. The lower panel displays columns representing densitometric analysis of the data (expressed as the BAG3/calregulin ratio) corresponding to the upper panel (*n* = 2). ^∗^*P* < 0.05, statistically significant differences, were calculated by Student's *t*-test for unpaired data. (c) Analysis of *bag3* mRNA levels by qRT-PCR. Fold induction of *bag3* mRNA levels (*y*-axis) in HUVEC controls and PEITC-treated cells is expressed relative to *β*-actin mRNA levels. Data are the mean values ± SD from two independent experiments performed in triplicate. ^∗^*P* < 0.05 and ^#^*P* < 0.01, statistically significant differences, compared to DMSO-treated cells (C), were calculated by one-way ANOVA with Dunnett's post hoc test using SigmaPlot12.0 software. (d) Phase-contrast images of HUVECs treated with (A) DMSO (control) at a final concentration (0.01%), with (B) PEITC (10 *μ*M) for 120 min, and with (C) PEITC (10 *μ*M) for 16 h. (D, E, F, G) HUVECs were transfected for 48 h with *bag3* siRNA (100 nM) or with a NT siRNA (100 nM) and treated with (D, F) DMSO (control) at a final concentration (0.01%) and with (E, G) PEITC (10 *μ*M) for 16 h. (e) HUVECs were transfected as described above. Total cell protein content was subjected to Western blot analysis to measure BAG3 levels and anti-calregulin antibody, as an internal loading control.

To assess whether JNK activation is involved in actin cytoskeleton modifications, 1 h before PEITC administration, HUVECs were pretreated with nontoxic concentration (10 *μ*M) of the specific JNK inhibitor SP600125 [34]. Phase-contrast observations revealed that the JNK inhibitor significantly reduced PEITC-induced morphological changes (Figure 3(a), A–D). Confocal analysis of actin staining indicated that pretreatment with the JNK inhibitor reverts cytoskeletal modifications induced by PEITC (Figure 4(a), E–H). The levels of phospho-JNK and phospho-c-Jun, a nuclear substrate of activated JNK, were detected by Western blot analysis to confirm the effectiveness of JNK inhibition. Activation of Rac1 in cells treated with PEITC in the presence of SP600125 confirmed that Rac1 acted upstream of JNK (Figure 3(b)). Taken together, these data strongly suggest the involvement of phospho-JNK in PEITC-mediated actin cytoskeleton remodeling.

### 2.4. PEITC Treatment Induces BAG3 Expression and Its Delocalization in HUVECs

BAG3 is the only member of the BAG family induced by stressful stimuli, mainly through the binding of the heat shock factor 1 to the *bag3* gene promoter [35] and also by JNK pathway activation [36]. BAG3 protein is also highly expressed in different tumor types [37–42] and regulates neoangiogenesis by interacting with and regulating ERK activity [20]. In addition, it has been demonstrated that BAG3 plays an important role in cell adhesion and cell migration [43] and is directly involved in maintaining actin folding [21]. Indirect immunofluorescence experiments were performed to investigate the cellular localization of BAG3 after PEITC treatment (Figure 4(a)). Analysis by confocal microscopy demonstrated that BAG3 in HUVECs, under physiological growth condition, appears to have a predominantly cytoplasmic localization; 2 h of PEITC treatment moved the protein in the protruding ends where it colocalized with F-actin at the cell membrane, as can be observed in the merged image (Figure 4(a), H). Taken together, these data demonstrated that BAG3 protein is subjected to changes in its expression and localization during PEITC treatment. Its delocalization to F-actin in proximity to cell membranes suggests a role of the BAG3 protein in cell morphology changes that have been observed.

To assess whether PEITC is able to induce changes in BAG3 protein levels, a protein expression analysis was performed. As shown in Figure 4(b), the BAG3 expression level increases after 16 h treatment. This result showed that PEITC also modulates BAG3 expression levels and its upregulation could be a direct consequence of JNK activation [36]. Furthermore, real-time PCR experiments have shown that the increase in the level of BAG3 protein correlates with the increase in the *bag3* mRNA (Figure 4(c)). Phase-contrast observations of HUVECs revealed that PEITC induced a dramatic change in cellular morphology after 120 min of treatment (Figure 4(d), A and B); however, prolonged exposure to PEITC (16 h) seems to show a recovery of cellular morphology similar to the control (Figure 4(d), A–C). This condition coincides with the increase in the expression of the protein observed in Figures 4(b) and 4(c). In order to demonstrate that BAG3 protein is directly involved in the recovery of cell morphology, we performed protein silencing experiments using specific siRNAs. As can be seen in Figure 4(d), E, when the BAG3 protein is silenced, the cells, after 16 h of treatment, do not show a recovery, whereas a nontargeted (NT) siRNA, used as a control, had no effect. Western blotting analysis in Figure 4(e) confirms silencing of the protein BAG3. These data show that BAG3 expression is essential for the recovery of cell morphology.

## 3. Materials and Methods

### 3.1. Reagents and Antibodies

Endothelial growth medium (EGM-2 BulletKit) was purchased from Clonetics (Walkersville, MD), and Vascular Cell Basal Medium (EGM Kit-VEGF) was purchased from ATCC. Phenethyl isothiocyanate (PEITC), SP600125, Hoechst 33342, protease inhibitor cocktail, and phalloidin-tetramethylrhodamine B isothiocyanate were purchased from Sigma (St. Louis, MO) and NSC-23766 from Merck Chemicals Ltd. (Darmstadt, Germany). Chemiluminescent detection reagents (ECL Plus Western Blotting) were purchased from Amersham Biosciences (Piscataway, NJ). LY294002 and antibodies to phospho-Akt (Ser473), Akt, SAPK/JNK, phospho-SAPK/JNK (Thr183/Tyr185), and phospho-FAK (Tyr397) were from Cell Signaling Technology (Danvers, MA); antibodies to BAG3 were from BIOUNIVERSA s.r.l (Montoro, AV, Italy), antibodies to PI3K, phospho-PI3K, and GAPDH from Santa Cruz Biotechnology Inc. (Santa Cruz, CA, USA), and anti-Rac1 antibodies from Millipore (Bedford, MA) and Pierce (Rockford, IL). Normal goat serum, horseradish peroxidase- (HRP-) conjugated secondary antibody, enhanced chemiluminescence reagents, anti-mouse IgG, and the Dylight 488-conjugated anti-mouse IgG were purchased from Jackson ImmunoResearch Laboratories (Suffolk, UK).

### 3.2. Cell Culture Conditions

Human umbilical vein endothelial cells (HUVECs) were purchased from the National Institute for Cancer Research (Genova, Italy). All experiments were performed on low-passage cell cultures. Cells were grown in EGM-2 or EGM Kit-VEGF at 37°C in a 5% CO_2_ atmosphere. Experiments were performed in the absence/presence of PEITC (10 *μ*M) and in the absence/presence of LY294002 (10 *μ*M), NSC23766 (100 *μ*M), and SP600125 (10 *μ*M) pretreatments, using 0.01% DMSO as a control.

### 3.3. Cell Viability Assay

The MTT (3-[4,5-dimetiltiazol-2,5-diphenyl-2H-tetrazolium bromide]) assay was used in order to assess the cell viability to compare the effect of the potential cytotoxicity of PEITC with a control condition. Such molecule is reduced by a mitochondrial enzyme succinate dehydrogenase to a formazan salt, which precipitates as blue/purple crystals. This is a colorimetric assay, in which the amount of formazan produced is measured spectrophotometrically and is proportional to the number of viable cells.

To perform the assay, the cells, from two different donors, were grown in 96-well plates, in numbers of 5 × 10^3^/cm^2^, and after 24 h were treated with increasing concentrations of PEITC from 2.5 *μ*M to 40 *μ*M in triplicate for 24 and 48 h. At the end of treatment, the plates were centrifuged at 1200 rpm for 5 min, the medium was removed, 100 *μ*l of 1 mg/ml MTT was added to each well, and the plates were kept at 37°C for the time necessary for the formation of salt formazan. The solution was then removed from each well, and the formazan crystals within the cells were dissolved with 100 *μ*l of DMSO. Absorption at 490 nm for each well was assessed by a Multiskan Spectrum Thermo Electron Corporation Reader. The data thus obtained were normalized with respect to the control and used to construct the dose-response histograms [44].

### 3.4. Flow Cytometry Analysis

Apoptosis was analyzed by propidium iodide incorporation in permeabilized cells and flow cytometry. Cells (5 × 10^3^/cm^2^) were cultured in 12-well plates. After 24 h, PEITC was added at the concentration of 10 *μ*M and cells were recultured for different times (24 h). For apoptosis analysis, permeabilized cells were labelled with propidium iodide (PI) by incubation at 4°C for 30 min with a solution containing 0.1% sodium citrate, 0.1% Triton X-100, and 50 mg/ml PI (Sigma-Aldrich, St. Louis, MO). The cells were subsequently analyzed by flow cytometry by a FACSCalibur flow cytometer (Becton Dickinson, North Ryde, NSW, Australia).

To evaluate cell cycle distribution, control and treated cells were harvested and nuclei were labeled with PI as described for apoptosis detection and analyzed by flow cytometry. Data from 2000 events per sample were collected, and the relative percentage of the cells in G0/G1, S, G2/M, and sub-G0/G1 phases of the cell cycle was determined using the ModFit software (Becton Dickinson, San Jose, CA, USA). Each determination was repeated three times [45].

### 3.5. Densitometry

Scanning densitometry of bands was performed with Image Scan (SnapScan 1212; Agfa-Gevaert NV). The area related to each band was determined using ImageJ software. Background was subtracted from calculated values. Results are expressed as mean of at least two separate experiments. Significance was determined by unpaired Student's *t*-test.

### 3.6. Western Blotting

Cells were harvested and immediately lysed with RIPA buffer (50 mM HEPES, 10 mM EDTA, 150 mM NaCl, 1% NP-40, 0.5% sodium deoxycholate, and 0.1% SDS (pH 7.4)) supplemented with protease inhibitor cocktail. For Western blot analysis, 20 *μ*g of the sample extract was resolved on 10–12% SDS-polyacrylamide gels using a minigel apparatus (Bio-Rad Laboratories, Richmond, CA). Nitrocellulose blots were blocked with 10% nonfat dried milk in Tris buffered saline with Tween 20 (TBS-T) (20 mM Tris-HCl (pH 7.4), 500 mM NaCl, and 0.01% Tween 20) and incubated overnight at 4°C with appropriate dilutions of primary antibodies with 10% (weight/vol) milk in TBS-T buffer or 5% bovine serum albumin. Immunoreactivity was detected by sequential incubation with an HRP-conjugated secondary antibody and enhanced chemiluminescence reagents according to routine protocols.

### 3.7. Rac1 Activity Assay

Rac1 activation assays were performed using a commercially available EZ-Detect Rac1 Activation Kit (Pierce, Rockford, IL). The assay uses a GST-fusion protein containing the p21-binding domain of p21-activated protein kinase 1 (PAK1) to selectively bind active Rac1 in the whole cell lysates. Lysates (500 *μ*g total protein) were incubated with glutathione Sepharose beads. Active Rac1 was collected on glutathione agarose beads and quantified by immunoblot analysis using an anti-Rac1 monoclonal antibody. On a separate immunoblot, total Rac1 from cell lysates was normalized and expressed as a ratio of total Rac1 to GAPDH.

### 3.8. Immunofluorescence

HUVECs were seeded onto 12 mm glass coverslips and grown for 24–48 h until 60–70% confluence. After treatment, cells were washed in PBS and fixed in 3.7% formaldehyde-PBS (30 min at room temperature). After washing, coverslips were treated for 10 min with 0.1 M glycine-PBS and permeabilized with 0.1% Triton X-100 for 10 min. After permeabilization, coverslips were washed again and incubated with a blocking solution (10% normal goat serum in PBS) for 1 h at room temperature. Staining of the actin cytoskeleton in fixed cells was performed with phalloidin-tetramethylrhodamine B isothiocyanate (0.05 *μ*g/ml) for 40 min at room temperature, followed by three rinses in PBS. Cells were labeled with an anti-BAG3 (3 *μ*g/ml) mouse monoclonal antibody AC-1 for 2 h at room temperature and Hoechst 33342 (2 *μ*g/ml) nuclear staining for 15 min. Coverslips were then washed 3 times in PBS, rinsed in distilled water, and glycerol-mounted on slides. A Zeiss LSM 510 Laser Scanning Microscope (Carl Zeiss MicroImaging GmbH) was used for data acquisition. To detect the nucleus, samples were excited with a 409 nm Ar laser. A 488 nm Ar or a 555 nm He-Ne laser was used to detect emission signals from target stains. A 63x (1.40 NA) Plan-Apochromat oil immersion objective was used. Images and scale bars were generated with Zeiss ZEN Confocal Software (Carl Zeiss, MicroImaging GmbH) and presented as a single stack. Images were processed using ImageJ software (NIH) and Adobe Photoshop CS version 5.0, and figures assembled using Microsoft PowerPoint (Microsoft Corporation).

### 3.9. mRNA Isolation and Real-Time RT-PCR

Total RNA isolation (mRNA) was performed by using the QIAzol Lysis Reagent (Qiagen). RNA (1 *μ*g) was reverse-transcribed by using the QuantiTect Reverse Transcription Kit (Qiagen). Real-time PCR was performed on LightCycler 480 (Roche) by using LightCycler 480 SYBR Green Master Mix, 2 *μ*l of cDNA (~50 ng), and the following primer pairs at the final concentration of 0.3 *μ*M: BAG3-human FW: 5′-CCTGTTAGCTGTGGTTG-3′ and BAG3-human RW: 5′-AACATACAGATATTCCTATGGC-3′ for the target gene and *β*-actin FW: 5′-AAAGACCTGTACGCCAACAC-3′ and *β*-actin RW: 5′-GTCATACTCCTGCTTGCTGAT-3′ for the housekeeping detection. Transcript quantities were compared using the relative Ct method, where the amount of the target normalized to the amount of the endogenous control (*β*-actin) and relative to the control sample is given by 2^(−ΔΔCt)^. The results are presented as mean ± SD of the mean of experimental triplicates. Significance was determined by one-way analysis of variance (ANOVA) with Dunnett's post hoc test using SigmaPlot12.0 software.

### 3.10. Transfections

HUVECs were transfected with a specific siRNA targeting *bag3* mRNA (50-AAGGUUCAGACCAUCUUGGAA-30) or a NT-siRNA (50-CAGUCGCGUUUGC GACUGG-30). For cell transfection, 5 × 10^3^ cells/cm^2^ were plated, in endothelial growth medium-2 media containing 2% fetal bovine serum and growth factors, to give 30–40% confluence. Cells were transfected with a final siRNA concentration of 100 nM using TransFectin (Bio-Rad Laboratories Inc., Hercules, CA, USA). Transfection efficiency was evaluated in each experiment by Western blot analysis.

## 4. Discussion

Cell proliferation is a tightly regulated process, organized by pro- and antiproliferative signals. Generally, the proproliferative signals are activated when new cells are required to replace damaged cells. Sustained proliferative signaling is a major characteristic of cancer cells, and PI3K/Akt activation is crucial for cell proliferation, while their PEITC-mediated inhibition is known to suppress cancer growth. PEITC inhibits Akt, a component of Ras signaling to inhibit tumor growth in several cancer types [13, 46]. PEITC is also known to inhibit EGFR and HER2, which are important growth factors and regulators of Akt in different cancer models [47, 48]. The inhibition of Akt may also be due to the suppression of EGFR or HER2 [47].

Overall, it is evident from these examples that PEITC can support the suppression of tumor cell growth through alternative pathways.

Unfortunately, so far, little is known about the activity of PEITC on normal cells, although in a contrasting manner our data are the first ones that try to clarify this activity in detail.

In order to study the activity of PEITC on the endothelial cells, we analyzed the PI3K/Akt pathway as described for tumor cells. However, surprisingly, the first results obtained conflicted with the initial hypothesis and with the literature. In fact, at the PEITC concentrations we used on HUVECs, we did not observe cell death but activation of the PI3K/Akt survival pathway. Moreover, the observation under a phase-contrast microscope showed significant morphological changes, such as volume reduction of cells at the very first minutes, while after 16 hours, cells appear again in their normal sizes without a significant loss in cell viability, even after 48 hours of treatment.

These observations have suggested to investigate the activity of Rac1, in the first minutes of PEITC treatment, which is known to be required to regulate actin polymerization and membrane protrusion [49], and we observed an increase in Rac1 activity and unexpectedly we also observed that PEITC induced an increase in total Rac1 protein.

Since JNK pathways are activated in response to a wide range of stimuli but most notably following cell exposure to a variety of biotic or abiotic stress events, such as infection, inflammation, oxidative stress, DNA damage, osmotic stress, or cytoskeletal changes [32], our studies show a significant increase in JNK activity.

Several studies showed that Rac1 activation was dependent on PI3K activity and that inhibitors of PI3K/Akt blocked Rac1 activation [23, 32]. Moreover, inhibition of the activity of Rac1 reduced JNK activity [50, 51]. In our study, the activity of Rac1 was abolished by the PI3K/Akt inhibitor LY294002, and the activity of JNK was abolished by the Rac1inhibitor NSC23766 suggesting that PI3K/Akt-Rac1 contributes to JNK activation.

Our results indicate that a key target of PEITC activity in HUVECs is represented by JNK. Indeed, inhibition of this kinase results in the abrogation of PEITC effects on the cytoskeleton. This finding is in line with the reported involvement of JNK in cytoskeleton remodeling [52].

JNK signaling led also to the activation of the c-Jun transcription factor, which is involved in the upregulation of several genes, among them, the BAG3 protein [36] that exerts an important role in endothelial cell survival and growth and in tumor neoangiogenesis [20]. Indeed, PEITC induces an increase in BAG3 levels in HUVECs. This hypothesis is supported by the fact that the increase in the expression of BAG3 corresponds to a recovery of cellular morphology after initial damage by PEITC treatment.

Downmodulation of the BAG3 protein levels by a specific siRNA resulted, after 16 hours of treatment, in the loss of recovery of the normal cellular morphology in respect to controls. These data suggest a role for BAG3 in the described events induced by PEITC.

BAG3 also utilizes its WW domain to engage in YAP/TAZ signaling. Via this pathway, BAG3 stimulates filamin transcription to maintain actin anchoring and crosslinking under mechanical tension and, by integrating tension sensing, ensures tissue homeostasis and regulates fundamental cellular processes such as adhesion, migration, and proliferation [53].

**Figure 5:**
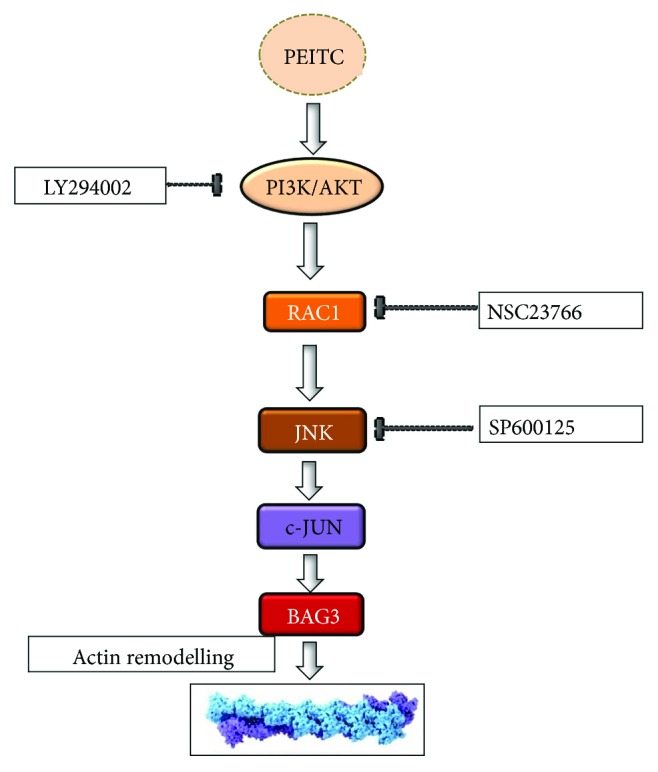
Schematic representation of the proposed model for PI3K/Rac1/JNK/c-Jun pathway activation and involvement of BAG3 as a target of the PEITC effect.

In conclusion, our results suggest that treatment with PEITC causes HUVEC damage to the cytoskeleton and induces activation of the PI3K/Akt-Rac1-JNK prosurvival pathway. c-Jun, a substrate of JNK, activates the transcription of *bag3* with a consequent increase in the BAG3 protein, which allows the refolding of actin and therefore the recovery of cell morphology (Figure 5).
